# Epigenetic factors and inflammaging: FOXO3A as a potential biomarker of sarcopenia and upregulation of DNMT3A and SIRT3 in older adults

**DOI:** 10.3389/fimmu.2025.1467308

**Published:** 2025-02-17

**Authors:** Diana Bogucka, Anna Wajda, Barbara Stypińska, Marcin Jerzy Radkowski, Tomasz Targowski, Ewa Modzelewska, Tomasz Kmiołek, Adam Ejma-Multański, Gabriela Filipowicz, Yana Kaliberda, Ewa Dudek, Agnieszka Paradowska-Gorycka

**Affiliations:** ^1^ Department of Molecular Biology, National Institute of Geriatrics, Rheumatology and Rehabilitation, Warsaw, Poland; ^2^ Department of Geriatrics, National Institute of Geriatrics, Rheumatology and Rehabilitation, Warsaw, Poland

**Keywords:** inflammation, ageing, sarcopenia, frailty, geriatrics, *FOXO3A*, *SIRT3*, *DNMT3A*

## Abstract

**Background:**

Epigenetic factors influence inflammaging and geriatric disorders such as sarcopenia and frailty. It is necessary to develop a biomarker/panel of biomarkers for fast and easy diagnostics. Currently, hard-to-access equipment is required to diagnose sarcopenia. The development of a biomarker/panel of biomarkers will prevent many older adults from being excluded from the diagnostic process.

**Methods:**

In this study, we analyzed selected gene expression profiles, namely, *SIRT1*, *SIRT3*, *SIRT6*, *DNMT3A*, *FOXO1*, *FOXO3A*, and *ELAVL1*, in whole blood. The study included 168 subjects divided into five groups: patients hospitalized at the Geriatrics Clinic and Polyclinic with sarcopenia, frailty syndrome, or without those disorders (geriatric control), and non-hospitalized healthy controls (HC) aged 25 to 30 years and over 50 years.

**Results:**

We revealed a lower mRNA level of *FOXO3A* (p<0.001) in sarcopenic patients compared to the geriatric controls. Furthermore, we detected upregulation of *DNMT3A* (p=0.003) and *SIRT3* (p=0.015) in HC over 50 years old compared to HC aged 25 to 30 years. Interestingly, we observed 2 cluster formations during the gene expression correlation analysis (*SIRT1*, *SIRT3*, *DNMT3A*, and *FOXO1*, *ELAVL1*). We also noted correlations of clinical parameters with mRNA levels in the sarcopenic patients group, such as vitamin D level with *SIRT1* (r=0.64, p=0.010), creatine kinase with *SIRT3* (r=–0.58, p=0.032) and *DNMT3A* (r=–0.59, p=0.026), creatinine with *DNMT3A* (r=0.57, p=0.026), erythrocyte sedimentation rate (ESR) with *FOXO3A* (r=0.69, p=0.004), and lactate dehydrogenase (LDH) with *FOXO3A* (r=–0.86, p=0.007). In the frailty syndrome group, we noted a correlation of appendicular skeletal muscle mass (ASMM) with *ELAVL1* (r=0.59, p=0.026) mRNA level. In the geriatric controls, we observed a correlation of serum iron with *FOXO3A* mRNA level (r=–0.79, p=0.036).

**Conclusions:**

Our study revealed *FOXO3A* as a potential biomarker of sarcopenia. Furthermore, we observed a high expression of epigenetic factors (*DNMT3A* and *SIRT3*) in older adults.

## 1 Introduction

According to the United Nations Department of Economic and Social Affairs (UN DESA), life expectancy is increasing and is projected to accelerate. By 2050, one in six people globally will be 65 years old or older (1). Therefore, a model of healthy aging with the absence of illness and physiological weakness is desirable. However, longevity does not necessarily align with a healthy lifespan. Extended years of life often coincide with chronic diseases, functional impairments, and reduced well-being (2). Musculoskeletal diseases are particular problems. Moreover, these diseases can have different pathological bases, such as those related to immunology or others associated with the overall nutritional status of the patients (3, 4). One of the entities within the scope of geriatrics and musculoskeletal diseases is sarcopenia which is associated with progressive loss of muscle mass and strength.

This clinical picture may be embedded into the frailty syndrome as well. However, frailty syndrome is a substantially broader concept, defined as “a medical syndrome with multiple causes and contributors that is characterized by diminished strength, endurance, and reduced physiologic function that increases an individual’s vulnerability for developing increased dependency and/or death” (5). A unified method for diagnosing frailty syndrome has not been yet established (6). Frailty syndrome includes physical, cognitive, and social aspects (7). Sarcopenia is often one of the determinants of the physical aspect of frailty syndrome. However, with regard to the broadness of the definition of frailty and the various diagnostic methods for these disorders, they may also occur independently (8, 9). Sarcopenia and frailty syndrome increase the risk of falls and fractures and are associated with the development of many diseases, including cardiovascular and respiratory diseases, and depression, anxiety and cognitive impairment. In a meta-analysis including 37 studies, it was revealed that sarcopenic patients have a 1.6x higher risk of falls and a 1.84x higher risk of fractures compared to patients without this condition (10). Regarding cognitive impairment, sarcopenic patients have a 2.25x higher risk of developing this symptom (11).

These conditions contribute to the loss of independence, institutionalization, social exclusion, a reduction in quality of life and premature death (7, 12–14). Sarcopenia is a complex geriatric disorder that requires expensive equipment such as DEXA (Dual Energy X-ray Absorptiometry) for diagnosis. Limited access to this device excludes many older adults from proper diagnostics and treatment.

The development of a biomarker/or a panel of biomarkers is necessary to provide fast, easy, and cost-effective diagnostic and disease progression monitoring. This approach aligns with the principles of “precision aging”, a growing field dedicated to personalizing healthcare for older adults by using biological markers to improve diagnosis and treatment (15). It will create an efficient tool for clinicians that contributes to demolishing barriers to diagnosing sarcopenia in the older population. Therefore, it is essential to find biomarkers for this disease in the blood to enable patients to be diagnosed based on a blood sample. The identification of a reliable, specific, and sensitive biomarker/panel of biomarkers in this material would allow it to be added to routine blood tests of older adults. This would significantly improve the detection of sarcopenia, shorten queues to specialists, as hard-to-find equipment would not be needed to detect the disease, and reduce patient frustration over the diagnostic process. When searching for biomarkers for sarcopenia, researchers focus on those associated with inflammatory processes (such as C-reactive protein, tumor necrosis factor, interleukin-8, and interleukin-6), hormones (including testosterone, dehydroepiandrosterone sulfate, and growth hormone), neuromuscular junction dysfunction (C-terminal agrin fragments), and metabolic changes (such as leucine and isoleucine) (16). Although several potential sarcopenia biomarkers have been identified, their accuracy and sensitivity limit their clinical use for diagnosis and monitoring. Based on the results of high-throughput studies of sarcopenia by other authors (17–19) and evidence of functional protein-protein interaction networks (**Supplementary Figure S1**), we selected several genes that are the subject of the present study and seem to be crucial in the sarcopenia development and may be helpful in further disease classification. Particularly, epigenetic changes play an important role in inflammaging, sarcopenia, and frailty (20, 21). Inflammaging is a theory suggesting that inflammation contributes to aging by decreasing the organism’s ability to manage stressors, leading to the progression of a chronic inflammatory state (22).

This was the reason for the selection of these specific genes responsible for methylation and acetylation, and genes from SIRTs’ molecular pathways in this study. A bidirectional interaction occurs between sarcopenia and systemic low-grade inflammation. Skeletal muscles secrete myokines and express membrane-bound factors that can modulate the immune system. Immune cells influence muscle function and induce muscle catabolism through changes in cytokine secretion, cell-cell interaction, and skeletal muscle infiltration (23). Therefore, we focused on genes associated with epigenetic modifications such as DNA Methyltransferase 3 A (*DNMT3A*), Sirtuin 1 (*SIRT1*), Sirtuin 3 (*SIRT3*), and Sirtuin 6 (*SIRT6*), and genes from SIRT’s molecular pathways [Forkhead Box O1 (*FOXO1*), Forkhead Box O3 (*FOXO3A*) and ELAV Like RNA Binding Protein 1 (*ELAVL1*)]. DNMT3A exerts an immunomodulatory function by methylating the promoter of the transcription factor 7 (*TCF7*) gene in T cells (24). Through the inhibition of pro-inflammatory pathways including Nuclear Factor Kappa B (NF-kB), Hypoxia-Inducible Factor 1-Alpha (HIF1a), Activator Protein 1 (AP-1), and p38 Mitogen-Activated Protein Kinase (P38MAPK), SIRT1 exhibits potent anti-inflammatory activity (25). SIRT3 is involved in both pro-inflammatory and anti-inflammatory responses (26). SIRT6 regulates inflammation through the Nuclear Factor Erythroid 2-Related Factor 2 (NRF2)-dependent signaling pathway (27). FOXO1 and FOXO3A immunoregulatory functions are modulated by Mammalian sterile 20-like kinase 1 and 2 (MST1 and MST2) (28). ELAVL1 regulates the mRNA stability of genes crucial for cytokine synthesis (29). Moreover, we undertook an analysis of the effect of age on the gene expression levels in healthy volunteers.

The main aim of this study was to recognize probable biomarkers related to sarcopenia. The hypothesis is that genes involved in epigenetic modifications, such as *SIRT1, SIRT3, SIRT6, FOXO1, FOXO3A, DNMT3A*, and *ELAVL1*, are critical for the development of sarcopenia and could be used as biomarkers for the diagnosis and monitoring of the disease.

## 2 Materials and methods

### 2.1 Patients

The present pilot study included 168 volunteers. They were divided into five groups: Sarcopenia Patients (N=15), Frailty Patients (N=36), Geriatric Controls (N=25), Healthy Controls over 50 years of age (N=41), and Healthy Controls 25 to 30 years of age (N=51). Patients with sarcopenia and frailty syndrome and those in the geriatric control group were enrolled at the Geriatrics Clinic and Polyclinic, National Institute of Geriatrics, Rheumatology and Rehabilitation in Warsaw, Poland. The geriatric control group consisted of patients hospitalized at this clinic in whom frailty syndrome and sarcopenia were not confirmed. After collection, the patients’ EDTA- blood was immediately transported to the laboratory. Volunteers’ blood from the healthy control groups (50+ and 25 to 30 years of age) was obtained from the blood donation facility. The blood was stored at -80°C for further analysis.

The selection of patients with sarcopenia was conducted based on the inclusion criteria defined by the European Working Group on Sarcopenia in Older People 2 (EWGSOP2) (8). Briefly, the criteria include the evaluation of muscle quantity [appendicular skeletal muscle mass (ASMM) measurement by dual-energy X-ray (DEXA) and calculated as ASMM/height^2^ with cut-off points for men of <7 kg/m^2,^ and <5.5 kg/m^2^ for women] and the assessment of muscle quality (measurement of grip strength by hand dynamometer with cut-off points for men of <27 kg and <16 kg for women).

A frailty index based on a standard comprehensive geriatric assessment (FI-CGA) was applied to the selection of patients with frailty syndrome (30). This test involves an evaluation of 10 domains: cognitive status, communication, mood, balance, mobility, bowel, bladder, nutrition, instrumental activity of daily living and activity of daily living (IADL and ADL), and social resources. Due to the broad definition of frailty syndrome and overlapping features of sarcopenia, in the present study patients classified to this group did not have sarcopenia and they were placed the frailty syndrome group based on the FI-CGA test.

Additionally, the severity of heart failure (HF) symptoms of hospitalized patients was classified, utilizing the four-stage New York Heart Association (NYHA) scale. Patients with HF classified in group I are asymptomatic and have no limitations in ordinary physical activity. Patients in group II have mild symptoms and slight limitations during ordinary activities. Group III is characterized by patients with marked limitation of activity due to symptoms and feel comfortable only at rest. Patients with HF in group IV experience severe restrictions and symptoms occur even at rest (31).

Exclusion criteria for the study were a history of hallucinatory disorders, schizophrenic or bipolar disorder, recent myocardial infarction or stroke (up to 4 weeks prior to study enrollment), and organ transplantation.

Before enrollment, each participant provided signed informed consent. The Research Ethics Committee of the National Institute of Geriatrics, Rheumatology, and Rehabilitation approved this study (approval number KBT-4/2/2018), which was conducted according to the Helsinki Declaration.

### 2.2 RNA extraction, quality check, reverse transcription

High-molecular-weight RNA was isolated from 500 ul of volunteers’ blood using a commercially available kit (Micro RNA Concentrator, A&A Biotechnology, Poland) according to the manufacturer’s protocol. RNA quality and quantity were assessed with a Quawell Q5000 spectrophotometer. The reverse transcription reaction was conducted using the High-Capacity cDNA Reverse Transcription Kit (Applied Biosystems, Thermo Fisher Scientific Inc.), according to the manufacturer’s protocol, and a thermocycler (Labcycler, Sensoquest).

### 2.3 Gene expression

The expression levels of seven genes were measured by qPCR reactions using TaqMan Gene Expression Master Mix (Applied Biosystems, Thermo Fisher Scientific Inc.) and TaqMan Gene Expression Assays (*SIRT1* - Hs01009006_m1, *SIRT3* - Hs00953477_m1, *SIRT6* - Hs00213036_m1, *FOXO1* - Hs01054576_m1, *FOXO3A* - Hs00818121_m1, *ELAVL1* - Hs00171309_m1, *DNMT3A* - Hs01027162_m1) (Applied Biosystems, Thermo Fisher Scientific Inc.). The qPCR reaction was conducted with a QuantStudio 5 Real-Time PCR System (Applied Biosystems, Thermo Fisher Scientific Inc.). Relative gene expression levels were calculated using the deltaCT method. The *GAPDH* gene (Hs02786624_g1) was selected as the most relevant housekeeping gene and was used to normalize the data.

### 2.4 Data analysis or statistics

Mean and standard deviations or median with first and third quartile were presented depending on variable distributions. Variable distributions were evaluated based on the Shapiro–Wilk test and histogram plot. For nominal variables, counts and percentage of observation with information (missing values were excluded) were shown. Differences between disease groups were assessed using Welch’s ANOVA, Kruskal–Wallis test, Pearson’s chi-squared test, or Monte Carlo test based on 2,000 replicates depending on the variable type and compliance with the assumption for parametric tests. If a significant difference was shown, *post hoc* pairwise analysis was performed, adjusting the p-value with the Bonferroni–Holm correction. Gene expression levels were shown on the log10 transformed axis for better visualization. Spearman correlations were calculated, and the rho coefficient and p-values were presented. Expression level differences between the two groups were calculated using the Mann–Whitney test.

Data preparation and calculations were performed using R language and environment [R Core Team (2021). R: A language and environment for statistical computing. R Foundation for Statistical Computing, Vienna, Austria. URL https://www.R-project.org/] and the following R packages: readxl (32), tidyverse (33), and naniar (34).

Figures were generated using the following R packages: ggstatsplot (35), cowplot (36), corrplot (37), ggpubr (38), Patchwork (39), and Scales (40). An expression correlation network was generated in Cytoscape 3.9.1 (41).

Missing data is summarized in the **Supplementary Material** (**Supplementary Table S1**).

## 3 Results

### 3.1 Patient characteristics

The gender ratio was balanced in each group (p = 0.670). Groups of healthy subjects (aged above 50 and 25 to 30 years) were specially selected to differ significantly in age from the patients in the other groups. The groups of hospitalized study subjects did not vary significantly by age (**Supplementary Figure S2**). The clinical characteristics of the geriatric control, frailty, and sarcopenia groups are summarized in **Table 1**.

**Table 1:** Clinical characteristics of the studied hospitalized groups.

	Geriatric control (N = 25)	Frailty group (N = 36)	Sarcopenia group (N = 15)	P value
Age (years)				
Mean ± SD	72.56 ± 7.86	80.50 ± 8.72	78.13 ± 12.37	ns*
Sex				
Female	17 (68.00%)	25 (69.44%)	9 (60.00%)	ns
Male	8 (32.00%)	11 (30.56%)	6 (40.00%)	
ASMM				
Mean ± SD	7.57 ± 1.04	7.19 ± 1.17	5.81 ± 0.85	<0.001
LDH (U/L)				
Median (1^st^ Q, 3^rd^ Q)	230.00 (215.25, 253.00)	231.00 (205.00, 261.00)	229.00 (212.00, 259.75)	ns
CK (U/L)				
Median (1^st^ Q, 3^rd^ Q)	72.00 (55.50, 106.75)	71.00 (45.25, 86.50)	68.00 (61.25, 72.75)	ns
**NYHA**				ns
0	23 (92.00%)	27 (75.00%)	9 (60.00%)	
I	0 (0.00%)	1 (2.78%)	0 (0.00%)	
II	1 (4.00%)	6 (16.67%)	4 (26.67%)	
II-III	1 (4.00%)	1 (2.78%)	2 (13.33%)	
Rheumatic Diseases				
Diabetes	3 (12.00%)	12 (33.33%)	2 (13.33%)	ns
Polyarticular degenerations	13 (52.00%)	18 (50.00%)	5 (33.33%)	ns
SLE	0 (0.00%)	3 (8.33%)	0 (0.00%)	ns
RA	2 (8.00%)	4 (11.11%)	3 (20.00%)	ns
Sjogren’s syndrome	0 (0.00%)	1 (2.78%)	0 (0.00%)	ns
Discopathy	8 (32.00%)	13 (36.11%)	6 (40.00%)	ns
Coxarthrosis	1 (4.00%)	3 (8.33%)	1 (6.67%)	ns
Gonarthrosis	3 (12.00%)	1 (2.78%)	3 (20.00%)	ns
SSc	0 (0.00%)	0 (0.00%)	1 (6.67%)	ns
Any Rheumatic Disease	19 (76.00%)	28 (77.78%)	10 (66.67%)	ns
Fasting blood glucose (mg/dl)				
Median (1^st^ Q, 3^rd^ Q)	94.00 (88.00, 100.25)	106.00 (87.00, 133.50)	89.00 (81.50, 95.00)	0.026
Vitamin D (ng/ml)				
Median (1^st^ Q, 3^rd^ Q)	34.02 (26.44, 42.74)	29.63 (20.41, 51.85)	34.32 (24.31, 37.75)	ns
Optimal	16 (64.00%)	17 (48.57%)	8 (53.33%)	
Suboptimal	4 (16.00%)	9 (25.71%)	5 (33.33%)	
Deficiency	5 (20.00%)	9 (25.71%)	2 (13.33%)	
Bone Diseases				
Osteopenia	3 (14.29%)	6 (20.00%)	8 (53.33%)	0.042
Osteoporosis	6 (28.57%)	10 (33.33%)	4 (26.67%)	ns
NT-proBNP (pg/ml)				
Median (1^st^ Q, 3^rd^ Q)	190.80 (122.25, 305.60)	231.10 (161.55, 438.85)	350.90 (248.05, 700.25)	ns
Elevated	12 (63.16%)	25 (80.65%)	13 (86.67%)	
Serum Fe (mcg/dL)				
Median (1^st^ Q, 3^rd^ Q)	96.50 (63.90, 129.30)	44.60 (32.60, 87.70)	85.00 (68.28, 89.58)	0.009
Optimal	7 (100.00%)	11 (64.71%)	7 (100.00%)	
Deficiency	0 (0.00%)	6 (31.58%)	0 (0.00%)	
Vitamin B12 (pg/mL)				
Median (1^st^ Q, 3^rd^ Q)	347.70 (278.90, 474.10)	357.90 (278.75, 503.02)	296.30 (267.80, 712.60)	ns
Optimal	20 (80.00%)	31 (86.11%)	14 (93.33%)	
Deficiency	3 (12.00%)	5 (13.89%)	1 (6.67%)	
ESR (mm/h)				
Median (1^st^ Q, 3^rd^ Q)	10.00 (8.00, 22.00)	22.00 (12.50, 35.25)	11.00 (8.00, 16.00)	0.029
Elevated	11 (44.00%)	27 (75.00%)	9 (60.00%)	
CRP (mg/dL)				
Median (1^st^ Q, 3^rd^ Q))	5.00 (5.00, 12.00)	7.00 (5.00, 25.50)	5.00 (5.00, 5.50)	0.022
Elevated	7 (28.00%)	14 (38.89%)	0 (0.00%)	
Albumins (g/dl)				
Mean ± SD	4.10 ± 0.32	3.97 ± 0.49	4.22 ± 0.36	ns
Decreased	2 (8.00%)	7 (20.00%)	0 (0.00%)	
Hemoglobin (g/dl)				
Mean ± SD	13.29 ± 1.43	12.11 ± 1.73	12.17 ± 1.68	0.014
Decreased	3 (12.00%)	12 (33.33%)	3 (20.00%)	
Creatinine (µmol/L)				
Median (1^st^ Q, 3^rd^ Q)	0.83 (0.71, 0.96)	0.85 (0.72, 0.95)	0.93 (0.73, 1.16)	ns
Elevated	3 (12.00%)	7 (19.44%)	2 (13.33%)	
Cholesterol (mg/dL)				
Mean ± SD	204.24 ± 42.99	173.00 ± 41.59	191.27 ± 38.57	0.026
Elevated	18 (72.00%)	11 (31.43%)	6 (40.00%)	
Decreased	3 (12.00%)	10 (28.57%)	4 (26.67%)	
LDL (mg/dL)				
Mean ± SD	120.50 ± 37.65	90.41 ± 30.27	94.99 ± 35.49	0.009
Elevated	15 (60.00%)	8 (22.86%)	6 (40.00%)	
Decreased	0 (0.00%)	1 (2.86%)	0 (0.00%)	
Triglycerides (mmol/l)				
Median (1^st^ Q, 3^rd^ Q)	135.00 (107.00, 188.00)	131.00 (88.50, 179.00)	83.00 (74.50, 126.00)	ns
Elevated	12 (48.00%)	12 (34.29%)	2 (13.33%)	
BMI [kg/m^2]				
Mean ± SD	29.86 ± 5.28	27.70 ± 6.44	22.87 ± 3.38	<0.001
TUG (s)				
Median (1^st^ Q, 3^rd^ Q)	14.00 (12.32, 15.00)	20.00 (14.00, 26.00)	13.34 (10.90, 17.50)	0.004
FI-CGA				
Mean ± SD	0.14 ± 0.06	0.36 ± 0.09	0.22 ± 0.10	<0.001

FI-CGA, Frailty Index Based on a Comprehensive Geriatric Assessment; TUG, Timed Up-and-Go scale (mobility scale); ASMM, appendicular skeletal muscle mass; LDH, lactate dehydrogenase; CK, creatine phosophokinase; NYHA class, New York Heart Association Functional Classification; NT proBNP, N-terminal pro B-type natriuretic peptide; Fe, iron; ESR, erythrocyte sedimentation rate; CRP, C-reactive protein; LDL, low-density lipoprotein; BMI, body mass index; 1st Q, lower quartile; 3rd Q, upper quartile; ns, not statistically significant; *, comparison also including healthy control groups (25 to 30 and 50+ years old).

In the present study, we observed elevated levels of fasting blood glucose (p=0.016), erythrocyte sedimentation rate (ESR) (p=0.022), and CRP (p=0.009) in the group of patients with frailty syndrome compared to the sarcopenic group. Furthermore, a reduced level of serum Fe (p=0.002), hemoglobin (p=0.020), and LDL (p=0.003) was detected in the frailty syndrome group compared to the geriatric controls. BMI level was significantly reduced in the group of patients with sarcopenia in comparison to the geriatric control (p<0.001) and frailty syndrome groups (p=0.006). The frailty syndrome group was characterized by a higher Timed Up and Go (TUG) value than the geriatric control (p=0.004) and sarcopenic groups (p=0.004).

### 3.2 Gene expression levels

**Figure 1:**
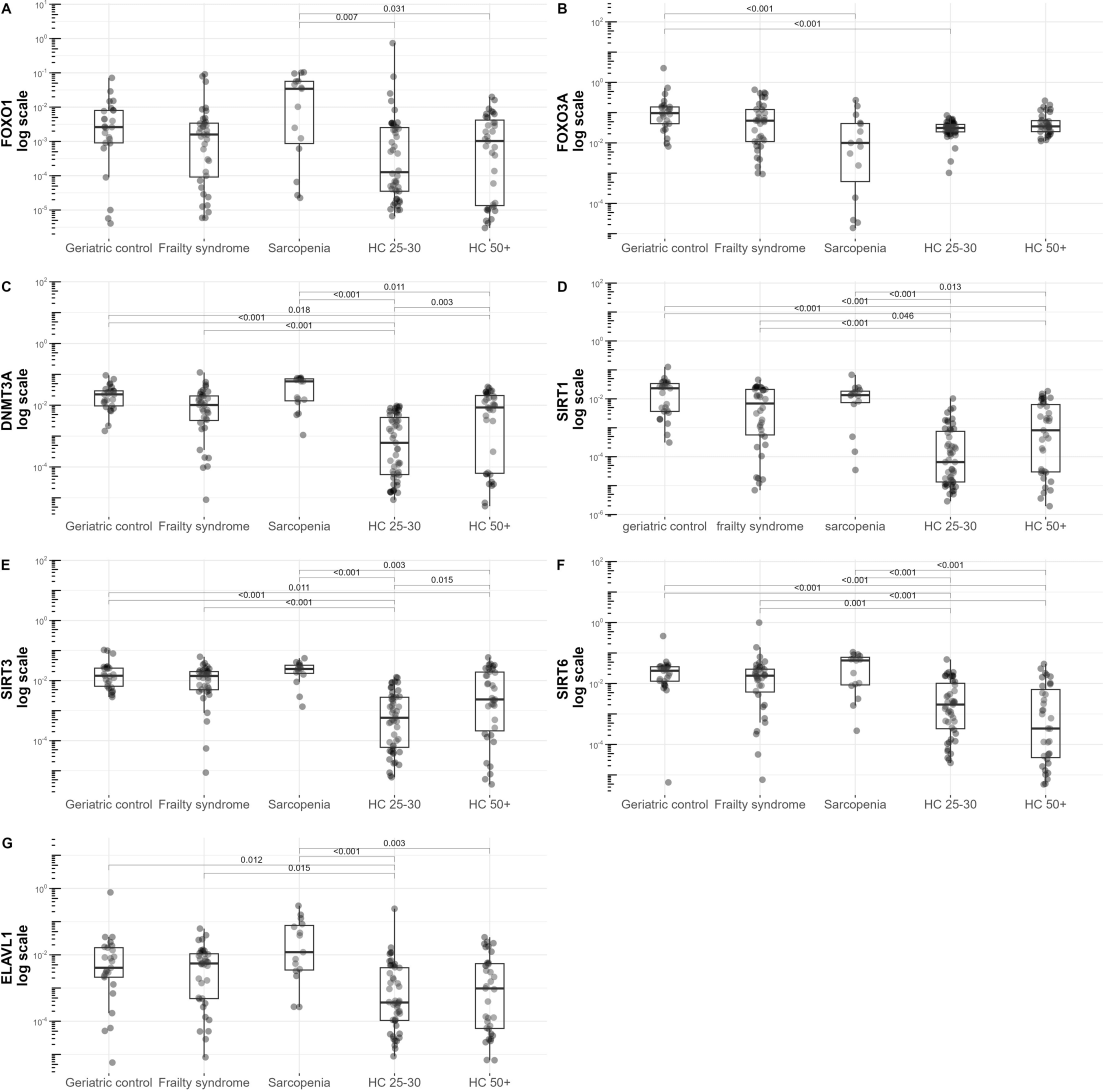
mRNA levels of **(A)** *FOXO1*, **(B)** *FOXO3A*, **(C)** *DNMT3A*, **(D)** *SIRT1*, **(E)** *SIRT3*, **(F)** *SIRT6*, **(G)** and *ELAVL1* normalized to the reference gene in the geriatric control group, frailty syndrome group, sarcopenic patients group, and healthy subjects 50+ years old (HC50+) and 25 to 30 years old (HC25-30). Data are presented on a logarithmic scale as boxplots with median and range.

Expression of *FOXO3A* was downregulated in patients with sarcopenia compared to all the analyzed groups. Moreover, the *FOXO3A* mRNA level was significantly lower in the sarcopenic group compared to the geriatric controls (p<0.001) (**Figure 1B**). mRNA levels of *DNMT3a* (p=0.003) and *SIRT3* (p=0.015) were significantly lower in the 25-to-30-year-old healthy control group in comparison to the 50+-year-old healthy control group (**Figures 1C, E**). This outcome may stem from the age impact. Additionally, we observed that more frequent low levels of *SIRT6* were observed in the older healthy controls (**Supplementary Table S2**). However, statistical significance in *SIRT6* expression between these healthy control groups (25 to 30 vs 50+) was not reached (**Figure 1F**).

We observed that the expression of the analyzed genes was more deregulated in patients with sarcopenia compared with other groups of patients and the healthy controls (25 to 30 years and 50+ years old). Significantly higher levels of *SIRT1* **Figure 1D**, *SIRT3, SIRT6, FOXO1,* **Figure 1A**, *ELAVL1* **Figure 1G**, and *DNMT3A* were detected in the sarcopenic group when compared to the 50+-year-old healthy control group. Interestingly, a clear grouping of patients into two clusters within the sarcopenia group by *DNMT3a* expression level was observed (**Supplementary Figure S3**). In patients with frailty syndrome, only *SIRT1* and *SIRT6* were significantly elevated compared to the 50+-year-old healthy controls (**Figure 1**).

### 3.3 Gene expression correlations

**Figure 2:**
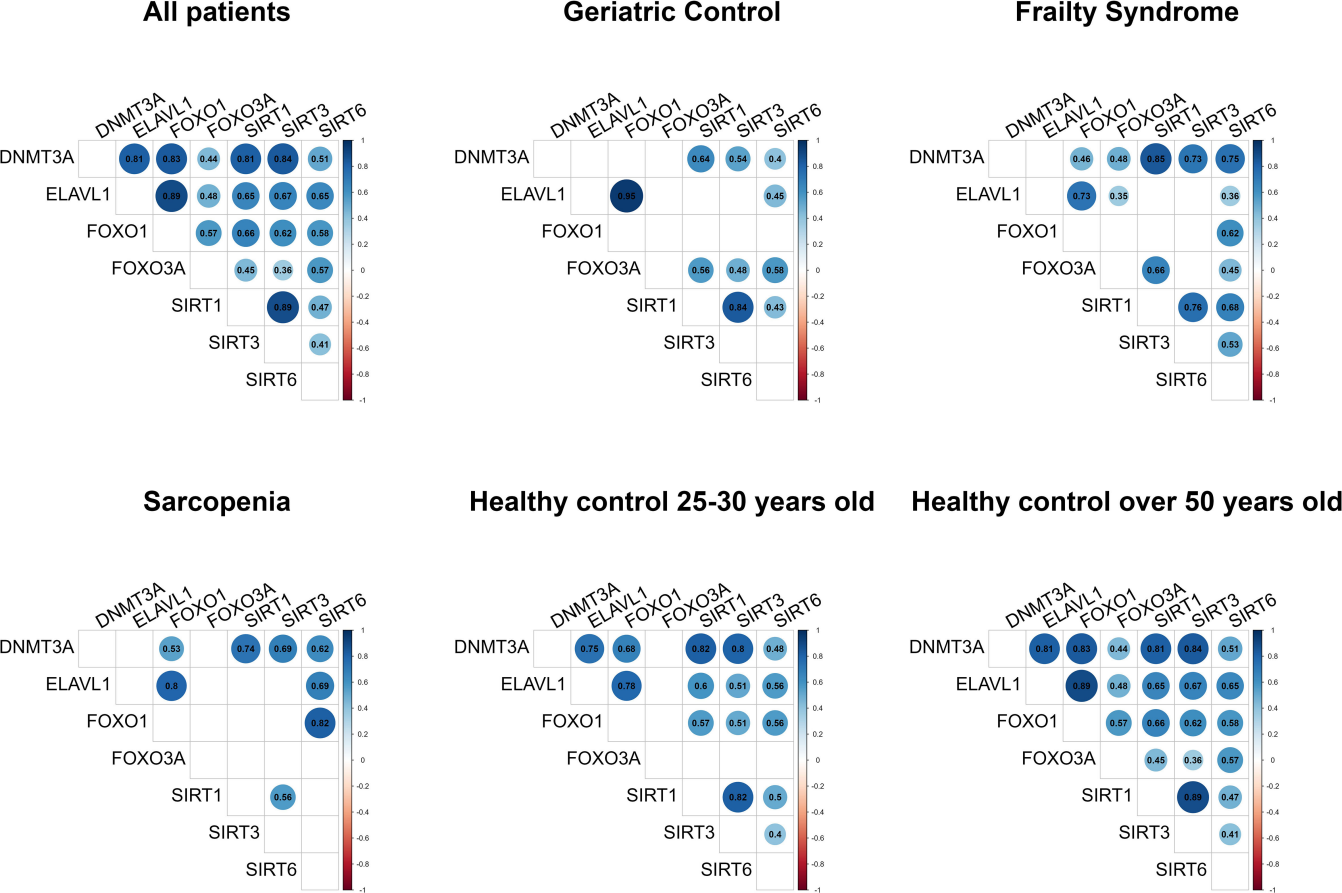
Gene expression correlation matrixes. Only statistically significant data are shown. Gene mRNA levels were normalized to the reference gene.

**Figure 3:**
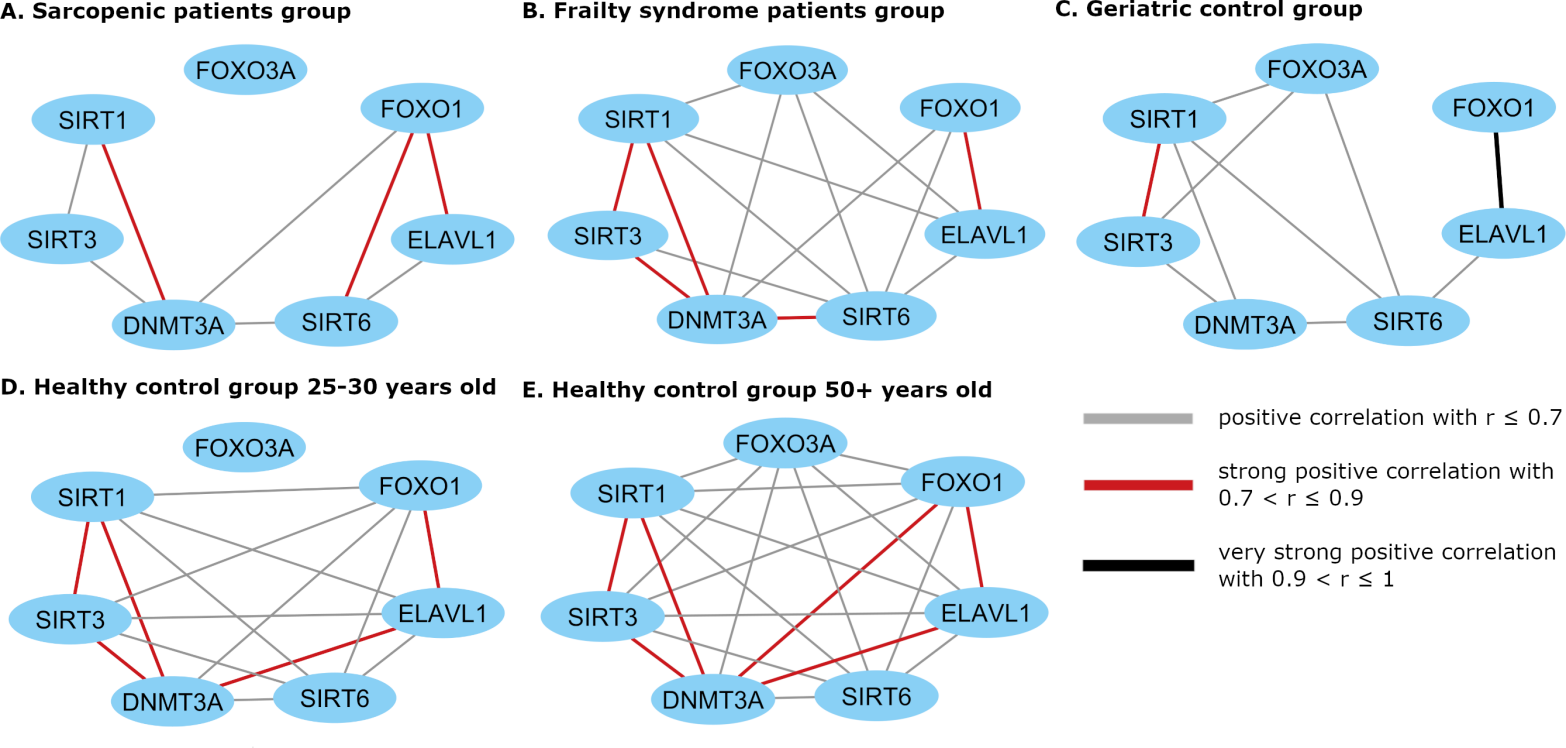
Cytoscape visualization of the expression correlation network in **(A)** sarcopenic patients group, **(B)** frailty syndrome group, **(C)** geriatric control group, and **(D)** healthy subjects 25 –30 years old and **(E)** 50+ years old. Gene mRNA levels were normalized to the reference gene.

Correlation matrixes present all statistically significant correlations for studied genes (**Figure 2**). Gene expression networks display correlations between mRNA expression profiles of each pair of genes (**Figure 3**).

Cluster formation was observed during gene expression correlation analysis. mRNA levels of *SIRT1*, *SIRT3*, and *DNMT3A*, and mRNA levels of *FOXO1* and *ELAVL1* were always correlated in every study group. In healthy subjects, a strong correlation between *ELAVL1* and *DNMT3A* was noted but not in the patient groups.

In the 50+-year-old control group, an expression correlation of each gene with every other studied gene was observed, while in the 25-to-30-year-old control group, correlations of all genes with each other were observed except for *FOXO3A*.

In patients suffering from sarcopenia, *FOXO3A* mRNA level did not correlate with other analyzed genes, while in the geriatric control group, *FOXO3A* expression correlated with the *SIRT* genes (*SIRT1* r=0.56, p=0.004; *SIRT3* r=0.48, p = 0.015; and *SIRT6* r=0.58, p=0.002). In the frailty group, *FOXO3A* mRNA level correlated with *SIRT1* (r=0.66, p <0.001), *SIRT6* (r=0.45, p =0.006), *ELAVL1* (r=0.36, p =0.035), and *DNMT3A* (r=0.48, p =0.003). Moreover, a strong correlation between *SIRT6* and *FOXO1* (r=0.82, p<0.001) mRNA levels was observed in the group of patients with sarcopenia, in contrast to the geriatric controls (r=0.35, p=0.089).

A significantly reduced number of pairs of correlated genes was observed in the geriatric control group (patients without sarcopenia and frailty syndrome) compared to the 50+-year-old healthy controls.

### 3.4 Gene expression correlation with clinic parameters

Iron level in blood was negatively correlated with *FOXO3A* level in the geriatric control group (r=–0.79, p=0.036).

**Figure 4:**
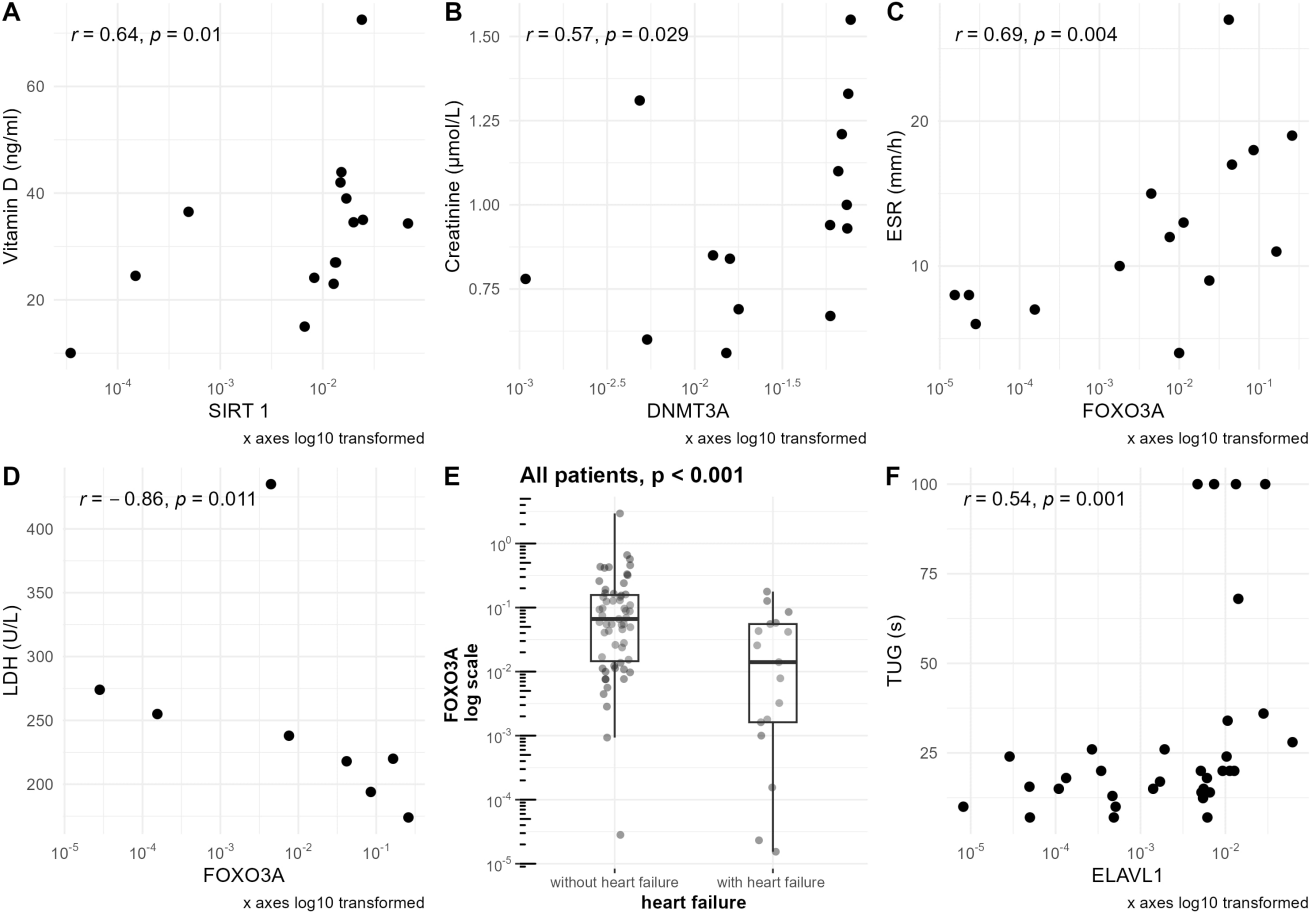
Associations of clinical parameters with mRNA levels of studied genes normalized to the reference gene. mRNA levels of the studied genes are presented on a logarithmic scale. **(A)** Correlation of vitamin D level with *SIRT1* mRNA level in the sarcopenic patients group. **(B)** Correlation of creatinine level with *DNMT3A* mRNA level in the sarcopenic patients group. **(C)** Correlation of ESR level with *FOXO3A* mRNA level in the sarcopenic patients group. **(D)** Correlation of LDH level with *FOXO3A* mRNA level in the sarcopenic patients group. **(E)** Differences in *FOXO3A* mRNA level in groups of patients with and without heart failure based on NYHA scale. For patients without heart failure, NYHA=0; for patients with heart failure, NYHA>0. Data are shown in a box plot with median and range. **(F)** Correlation of TUG with *ELAVL1* mRNA level in the frailty syndrome group.

Vitamin D level was positively correlated with *SIRT1* mRNA level in sarcopenic patients (r=0.64, p=0.010) (**Figure 4A**), and *SIRT6* mRNA level in the geriatric control group (r=0.44, p=0.027) (**Supplementary Figure S3A**).

No statistically significant correlations were observed when analyzing the B12 vitamin level with all the studied genes separately.

**Figure 5:**
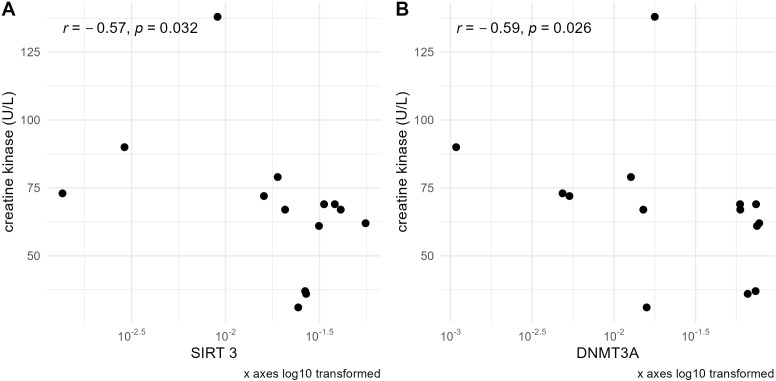
Correlations of serum creatine kinase concentration with **(A)** *SIRT3* and **(B)** *DNMT3A* mRNA levels normalized to the reference gene in the sarcopenic patients group. mRNA levels of the studied genes are presented on a logarithmic scale.

In the group of sarcopenic patients, negative correlations of serum creatine kinase concentration with the expression of *SIRT3* (r=–0.57, p=0.032) and *DNMT3A* (r=–0.59, p=0.026) were observed (**Figure 5**).

Furthermore, *DNMT3A* was also positively correlated with creatinine levels in this group (r=0.57, p=0.029) (**Figure 4B**).

**Figure 6:**
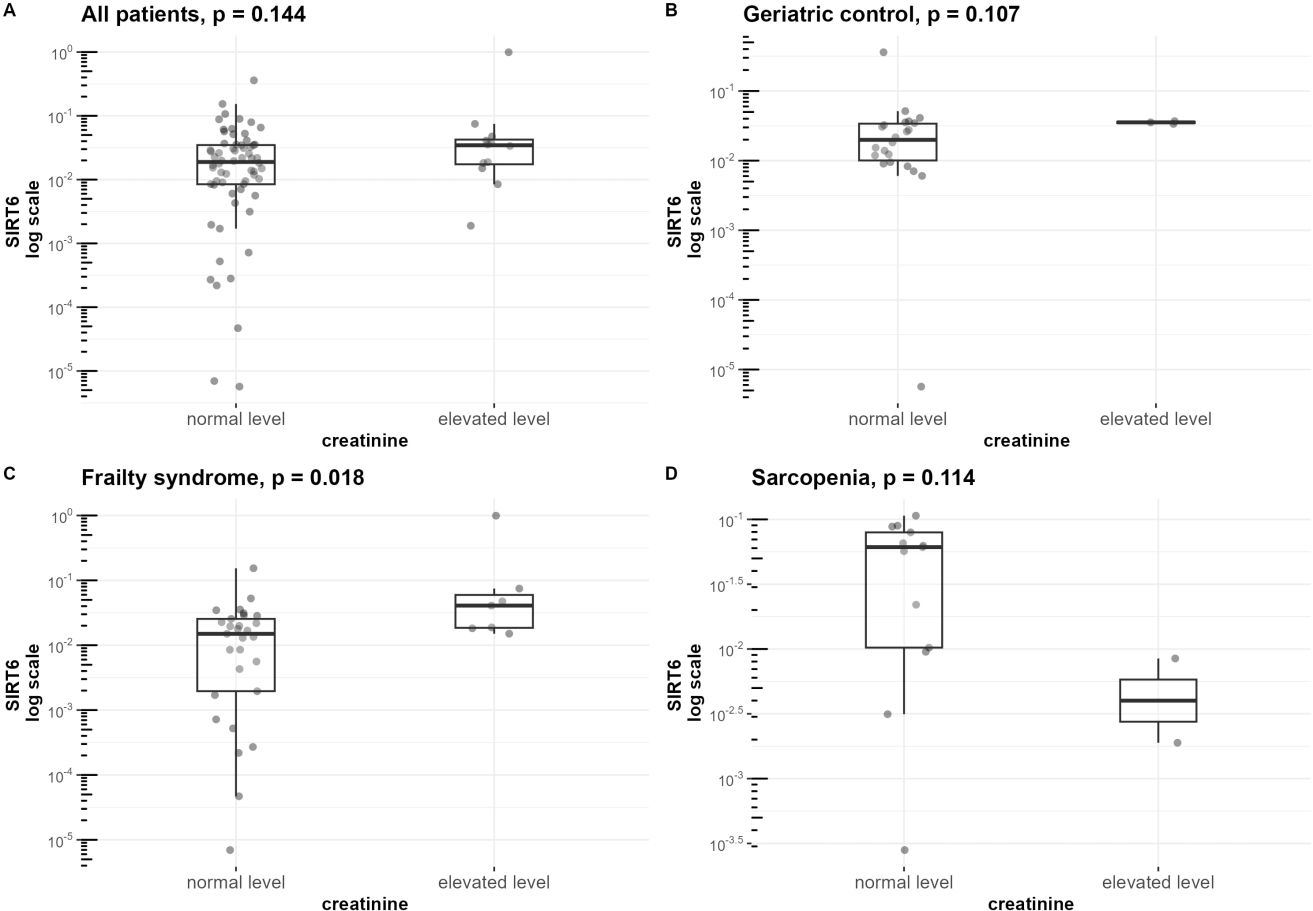
Differences in *SIRT6* mRNA level normalized to the reference gene in groups of patients with normal and elevated creatinine levels. mRNA levels of *SIRT6* are presented on a logarithmic scale. Data are shown in a box plot with median and range. **(A)** All three hospitalized groups combined (geriatric control group, frailty syndrome group, and sarcopenic group), **(B)** geriatric control group, **(C)** frailty syndrome group, and **(D)** sarcopenic group.

Creatinine concentration was additionally correlated with *FOXO3A* (r=0.51, p=0.009) (**Supplementary Figure S3B**) and *SIRT6* (r=0.42, **Figure 6B**) in the geriatric control group. When analyzing all the hospitalized groups together, a statistically significant but weak correlation between creatinine and the expression of *SIRT6* (r=0.31, **Figure 6A**) was observed. In patients with frailty syndrome, those with elevated creatinine levels in serum were characterized by significantly higher expression of *SIRT6* compared to those with lower levels (p=0.018) (**Figure 6C**). Furthermore, we detected a correlation between serum creatinine and vitamin D levels in the frailty syndrome group (r=0.36, p=0.036) (**Supplementary Figure S3C**).

**Figure 7:**
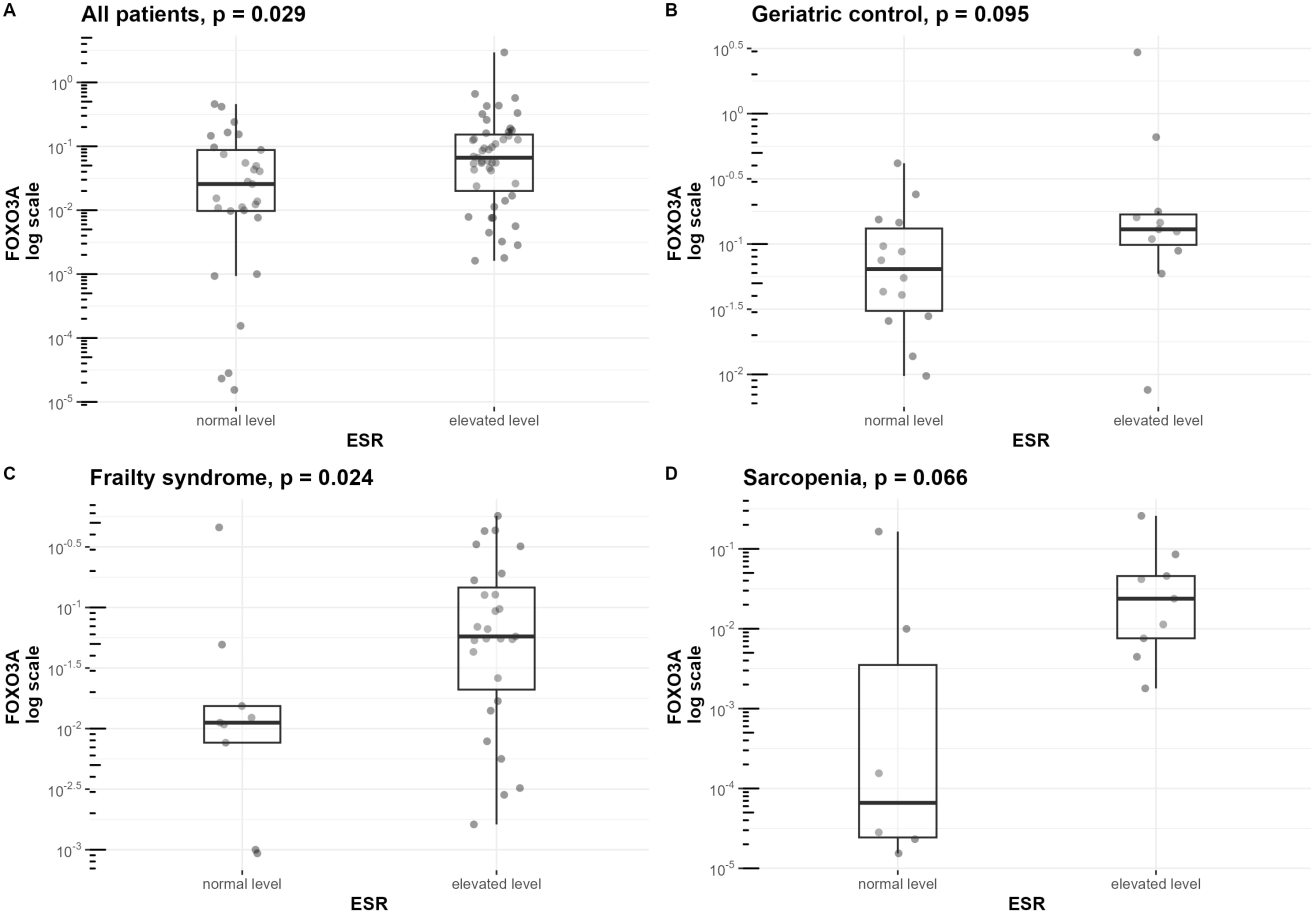
Differences in FOXO3a mRNA level normalized to the reference gene in groups of patients with normal and elevated ESR. mRNA levels of FOXO3A are presented on a logarithmic scale. Data are shown in a box plot with median and range. **(A)** All three hospitalized groups combined (geriatric control group, frailty syndrome group, and sarcopenic group), **(B)** geriatric control group, **(C)** frailty syndrome group, and **(D)** sarcopenic group.

ESR was correlated with the expression of *FOXO3A* in the sarcopenia patients group (r=0.69, p=0.004) (**Figure 4C**), geriatric control group (r=0.41, p=0.042) (**Supplementary Figure S3D**), and when analyzing all hospitalized groups together (r=0.38, p<0.001)(**Supplementary Figure S3E**). Furthermore, the upraised mRNA level of *FOXO3A* in the group of patients with elevated ESR level was observed in the frailty syndrome group (p=0.024) (**Figure 7C**), and when analyzing all hospitalized groups together (p=0.029) (**Figure 7A**). Similar trends, but not statistically significant, were observed in sarcopenic patients group and geriatric control group (**Figures 7D, B**).

Lactate dehydrogenase (LDH) was negatively correlated with *FOXO3A* mRNA level (r=–0.86, p=0.011) only in the sarcopenic patients group (**Figure 4D**).

When analyzing all the hospitalized patients together, a significant difference in *FOXO3A* mRNA level was observed between patients without heart failure (NYHA 0) in comparison with the patients with different stages of heart failure (NYHA >0) (p<0.001). Patients with heart failure had lower mRNA levels of this gene (**Figure 4E**).

N-terminal pro-B-type natriuretic peptide (NT-proBNP) concentration was correlated with *DNMT3a* mRNA level (r=0.52, p=0.049) only in the group of patients with sarcopenia (**Supplementary Figure S3F**). When analyzing all hospitalized groups together, NT-proBNP was correlated with *SIRT6* mRNA level (r = 0.26, p = 0.034).

The development of rheumatic disease was associated with a lower level of *DNMT3A* in the frailty patients group (p=0.002) (**Supplementary Figure S5**).Furthermore, in this group, a correlation between appendicular skeletal muscle mass (ASMM) and the expression of *ELAVL1* (r=0.59, p=0.026) was observed. However, in 61% of the patients with frailty syndrome the ASMM parameter was missing data.

Additionally, in the frailty patients group, FI-CGA value was correlated with the expression of *ELAVL1* (r=0.37, p=0.027) (**Supplementary Figure S4G**) and *FOXO3A* (r=0.34, p=0.045), and TUG value was correlated with *ELAVL1* (r=0.54, p=0.001) (**Figure 4F**) and *FOXO1* (r=0.35, p=0.043).

## 4 Discussion

Sarcopenia and frailty syndrome are both geriatric conditions. In sarcopenia, loss of muscle mass and strength is an essential factor, while frailty syndrome includes cognitive, physical, and social aspects. There is a partial overlap in the clinical picture of those conditions, and patients with sarcopenia may develop some, or all, of the symptoms of patients with frailty. Likewise, patients with frailty may or may not develop sarcopenia. Due to the strong association between these conditions, their pathogenesis may be at least partially similar. This may be the reason for the absence of differences in gene expression profiles between the studied groups of patients.

Currently, there is no registered drug for sarcopenia which is due to not fully understanding the pathogenesis of this disease. Our pilot research may contribute to an understanding of signaling pathways in this disease, which may lead to the overcoming and prevention of the disorder. Moreover, we focused on particular pathways associated with epigenetic regulations to avoid informative noise and combine results with the clinical picture of the patients.

In this study, we present a potential candidate for a novel blood biomarker of sarcopenia - *FOXO3A*. Furthermore, we detected upregulation of the mRNA levels of important epigenetic enzymes, *DNMT3A* and *SIRT3*, in the whole blood of healthy controls over 50 years old, compared to healthy controls 25 to 30 years old. This finding may indicate their importance in the molecular aspects of the aging process.

Our study differs from previous approaches in methodology. Instead of using a group of healthy older adults as a control group for identifying biomarkers of sarcopenia in whole blood, we selected a group of patients where sarcopenia was suspected. This methodology highlights the even greater value of the discovered marker. It is worth noting that patients from the sarcopenic group did not differ statistically by age (the average age of the sarcopenic patients was 78.13 ± 12.37, and the geriatric controls was 72.56 ± 7.86). Additionally, our study also included two groups of healthy, non-hospitalized volunteers aged 25 to 30 and over 50. Therefore, the design of the experiment also enabled the investigation of the influence of age on the expression of the studied genes.

### 4.1 *FOXO3A* as a potentially prognostic marker

In the present study, we observed lower expression of *FOXO3A* in the blood of patients with sarcopenia compared to the geriatric controls, which may be related to systemic inflammation. However, further research is needed to confirm it. . Interestingly, the decreased level of *FOXO3A* was also noted in healthy blood donors compared to the geriatric controls, possibly due to elevated levels of inflammatory cytokines in the blood donors (42). Thus far, we have not found in the available literature comprehensive *FOXO3A* expression analysis in the blood of sarcopenia patients in comparison to geriatric patients or patients who suffer from frailty syndrome.

FOXO3A is a transcription factor involved in several biological processes, including oxidative stress resistance and inflammation (43). It plays an important role in immunoregulation (44), including suppression of cytotoxic CD8+ T cell responses (44) and promoting conversion of naïve CD4+ T cells to regulatory T cells (Tregs) (28), which play an anti-inflammatory role (45) and are also involved in muscle regeneration (46). This geroprotective gene is implicated in ageing and healthspan, promoting longevity (47). Moreover, studies have shown that *FoxO3* ^−/−^ mice displayed signs of premature aging of the enteric nervous system (48), and FOXO3Adownregulation was also reported in other inflammatory diseases, such as ankylosing spondylitis (43), chronic obstructive pulmonary disease (COPD), and ulcerative colitis (UC) (49). In aged non-human primate skeletal cells, *FOXO3A*was identified as a hub gene for skeletal muscle homeostasis (50). However, constitutional activation of *FOXO3A* led to muscle atrophy viaincreased Atrogin-1 expression in a mouse C2C12 cell model (51). Notably, FOXO3A‘s role may differ between primates and mice, due to the primate-specific muscular isoform FOXO3A-Short, primarily expressed in muscles (52). These findings suggest that FOXO3A may be useful for sarcopenia not only as a biomarker but also as a medical target, as regulation of the AKT/TOR/FOXO3A signaling pathway can ameliorate this entity (53, 54).

In this study, we observed that the expression level of *FOXO3A* correlated with *SIRT1*, *SIRT3*, and *SIRT6* in geriatric controls, and *SIRT1*, *SIRT6*, *FOXO1*, *ELAVL1*, and *DNMT3A* in frailty syndrome. In contrast, no correlations were found between *FOXO3A* and other studied genes in the sarcopenia group. Together with the reduced expression level of FOXO3Ain the sarcopenia group, this suggests an alteration in the *FOXO3A* molecular pathway in peripheral blood cells. Furthermore, we observed increased expression of *SIRT3*, *SIRT6*, *FOXO1*, and *ELAVL1* in patients with sarcopenia compared to the geriatric controls and the frailty group, although these differencesdid not reach statistical significance. These observations may point to changes associated with the SIRTs pathway. However, this hypothesis requires further verification and additional studies to confirm the involved molecular pathway.

Furthermore, the molecular pathway of FOXO3A may be also influenced by age-related factors. No correlation between *FOXO3A* and the studied genes in the 25-to-30-year-old healthy control group, while in the 50+-year-old healthy control group, correlations were detected with all the tested genes.

Interestingly, we noticed that patients with an elevated ESR also had elevated *FOXO3A* mRNA levels. This finding aligns with the research of Wang et.al., whodescribed a correlation between *FOXO3A* serum expression and ESR level in both active and remissive rheumatoid arthritis patients (55). This outcome may support the hypothesis that reduced FOXO3A levels are associated with inflammatory processes and inflammaging. However, it should be noted that we were unable to conduct ESR tests in a group of healthy patients, so this hypothesis needs confirmation in further studies. Additionally, ESR level is affected by various factors such as age, sex, smoking, alcohol consumption, physical activity, obesity, and related metabolic syndrome, all of which should be consideredin future investigations (56).

In our study, we found a strong negative correlation between *FOXO3A* mRNA level and the iron serum concentration in the geriatric control group. However, the missing data in this group must be taken into consideration during the interpretation of the results. Nevertheless, these results are consistent with previous studies linking *FOXO3A* expression and iron metabolism. Xia et al. demonstrated that FOXO3A can counteract the deleterious effects of iron overload in osteoblast cells (57). Iron overload is a major event in ferroptosis, an iron-dependent type of cell death (58). Furthermore, Zhao et al. revealed that FOXO3A can inhibit ferroptosis in ATDC5 chondrocytes (59). Dysregulation of iron homeostasis with age is thought to play an important role in the pathogenesis of age-related diseases and increased mortality (60).

### 4.2 *SIRT3* and its impact on aging

The next analyzed gene in this study, which seems important for understanding sarcopenia’s pathogenesis, is SIRT3. SIRT3 is an NAD^+-^dependent mitochondrial deacetylase primarily located in mitochondria, where it regulates metabolism and stress response. However, SIRT3 regulation and sensitivity to changes in activity are tissuespecific and the type of stress also influences the SIRT3 signaling pathway (61). Notably,SIRT3 is associated with both pro-inflammatory and anti-inflammatory activities, which may explain inconsistencies in the literature regarding its role (26).

In the present study, we observed elevated *SIRT3* mRNA levels in the whole blood of the healthy control group over 50 years old compared to those aged 25 to 30. This increase may be linked to reactive oxygen species (ROS) production and aging (62, 63). The difference in *SIRT3* expression between the younger and older controls may be explained by the fact that younger people have better functioning mechanisms related to stress reduction. Various stressors are known to elevate SIRT3 levels (61). Perhaps different activities, multimorbidity, and pharmacotherapy may also have an impact on the *SIRT3* expression. Another possibility is that other genes , e.g., SIRT2, may compensate for SIRT3 function in younger individuals. Heinonen et al. demonstrated that silencing both SIRT3 and SIRT2 caused an increase in cytokine secretion, whereas silencing only one of these genes caused no significant change, suggesting functional redundancy between these genes (26). It is also important to notethat *SIRT3* mRNA upregulation does not necessarily translate to increased enzyme activity, as sirtuins require the NAD+ cofactor for function.. NAD+ levels, however, are tissue- and compartment-specific and remain poorly characterized in human aging (64).

The hypotheses mentioned above regarding the increase of SIRT3 expression level with age require further investigation. Moreover, sirtuins‘ ability to compensate for each other due to their mobility and similar features suggests that targeting multiple sirtuins or the entire group may be a more effective drug development strategy (26). However, tissue and context specificity of SIRT3 should be carefully considered in pharmacodynamics of newly designed drugs. Although the upregulation of *SIRT3* in the whole blood in the older group was reported, due to its tissue and context specificity, there are also findings that in the muscles of older humans, the SIRT3 level can be downregulated. Furthermore, upregulation of SIRT3 was shown in caloric restriction and exercise demonstrating its pro-longevity feature (65). That is why upregulation of SIRT3 was recently proposed to prevent aging. However, it is worth noting that this upregulation may be caused by stressors. Our study demonstrated, that systemic upregulation of SIRT3 proposed to prevent aging should not be considered as a new drug target due to its complex nature, tissue specificity and observation that older humans in physiological aging already have *SIRT3* upregulated in the blood.

### 4.3 *DNMT3A*, inflammation, and creatinine levels

Currently, it is well-known that epigenetic factors are important in aging and chronic inflammation, including sarcopenia (66). DNMT3A, an epigenetic enzyme involved in gene silencing via cytosine methylation (67), plays a key role in stabilizing CD4^+^ T cells and regulating early effector CD8^+^ T cells differentiation (24).

In our study, *DNMT3A* level was upregulated in the healthy controls over 50 years old, compared to those aged 25to 30 years. Similar results were described in the liver tissue of older adults (56 to 78 years) compared to the younger group (16 to 48 years),while other studies observed declining DNMT activity or expression in aged fibroblasts and individuals over 80 (68–70). Based on these studies and our outcomes, *DNMT3A* mRNA level might increase with age and then decline at a certain point. The average age of the 50+ control group in our study was 55, while Midic et al. examined patients over 80 years old, which may explain this inconsistency. Moreover, the younger group that was used for comparison with older patients had a wide age range (20–40) and was similar in upper age limit to our group of older patients. However, expression of DNMT3A and activity patterns associated with age have not been completely understood and require further investigation.

In the sarcopenia group, two clusters of patients with high and low expression of the *DNMT3A* gene were observed. Patients with elevated *DNMT3A* levels also showed higher creatinine levels, though thecorrelation was weak, suggesting independent activation of both factors. Neto et al. demonstrated that serum creatinine is elevated in inflammaging (71). It should be notedthat in our study creatinine level was slightly elevated in sarcopenic group, however, that observation was not statistically significant. Furthermore, upregulation of creatinine in blood is not always indicative of kidney impairment. Other factors e.g., vitamin D receptor activation, can increase serum creatinine levels without influencing the glomerular filtration rate (72). In conclusion, the link between DNMT3A levels and creatinine levels may reflect varying inflammation levels in sarcopenic patients, but further studies are needed.

### 4.4 Clinical associations

Referring to the effects of vitamin D, other research demonstrated that its supplementation may upregulate SIRT1 and SIRT6 (73, 74). This is consistent with our observation of the correlation between vitamin D and SIRT1 expression in the sarcopenic group and SIRT6 in the geriatric control group. Furthermore, we noted a correlation between vitamin D and serum creatinine in the frailty group, where patients withelevated creatinine also exhibited an increased *SIRT6* expression. This aligns with the previously found elevated creatinine level following vitamin D receptor activation (72).

Cardiac diseases are strongly associated with sarcopenia (75). The intensity level of HF is assessed with NYHA functional classification (76). In this study, 40% of sarcopenic patients were classified as NYHA II or II-III,however, the result was not statistically significant. Sarcopenia and HF are considered co-morbidities according to the task force for the diagnosis and treatment of acute and chronic heart failure of the European Society of Cardiology (ESC) (76).A meta-analysis reported sarcopenia prevalence among HF patients as high as 34% (77, 78). This may be related to the interplay between HF and muscle wasting. Heart failure can favor sarcopenia through hormonal changes, malnutrition, release of cytokines, impaired mitochondrial function, and others (79, 80). Mangner et al. demonstrated that chronic heart failure impairs antioxidative and metabolic responses and activates protein degradation pathways in the quadriceps (81). Moreover, sarcopenia can promote HF, *inter alia*, by pathological ergo reflex (79, 80).

NT-proBNP, a marker of cardiac dysfunction, is upregulated during inflammation (82). With reference to the earlier discussion on DNMT3A, its upregulation during T-cell activation leads to inflammation, which may be connected to our other finding—a significant correlation between NT-proBPN level and DNMT3A expression in the sarcopenia group.

Creatine kinase (CK), a marker of myocardial infarction, muscular dystrophy, and cerebral diseases, may inversely correlate with skeletal muscle, or cardiac or brain impairment. Interestingly, in our study, SIRT3 and DNMT3A mRNA levels showed a negative correlation with CK in the sarcopenic patients. This aligns with a previous study showing a negative correlation between SIRT3 protein levels and plasma CK-myocardial band (CK-MB) in rats (83).

LDH, another predictor of cardiovascular incidents (84), was strongly negatively correlated with *FOXO3A* mRNA level in sarcopenia patients. Additionally, *FOXO3A* levels were higher in patients classified as NYHA 0 compared to those classified as NYHA>0 when analyzing all the hospitalized patients together.

### 4.5 ELAVL1 and muscle metabolism

The last gene analyzed in the present study is ELAVL1. ELAVL1, a Human antigen R (HuR), is a posttranscriptional regulator of gene expression that binds to the adenylate-uridylate-rich elements (ARE) in 3’ untranslated regions (3′UTR) in mRNA and stabilizes the transcript (85). HuR expression is elevated in activated T cells, whereit promotes T cell CD4+ activation and Th2 and Th17 differentiation, affecting immune responses (86). In our study, *ELAVL1* was elevated in the sarcopenia group compared to frailty syndrome and geriatric control groups. *ELAVL1* was correlated with FI-CGA and TUG in the frailty syndrome group, suggesting a link between inflammation and functional mobility. FI-CGA is a scale used to assesses frailty, with higher scores indicating poorer outcomes (87). The TUG test evaluates overall functional mobility, where higher scores (overall time of the test) are associated with poorer performance. Lasselin et al. demonstrated that inflammation increases the TUG score (88). It is possible that *ELAVL1* might be correlated with TUG and FI-CGA as a result of inflammation, however, the underlying character of these associations requires further investigation.The stronger correlation with TUG may reflect its direct association with mobility, unlike FI-CGA, which incorporates cognitive and social factors. However, we also detected a correlation between ELAVL1 mRNA level and ASMM in the frailty syndrome group, though missing data of ASMM in this group limits the conclusions. The role of ELAVL1 in skeletal muscle metabolism and flexibility has been linked to lipid metabolism, as shown by Mynatt et al., who observedelevated expression of ELAVL1-dependent genes in skeletal muscles of metabolically flexible patients. Furthermore, they proved altered lipid metabolism in skeletal muscle-specific *Elavl1* deficient mice (89). Conversely,Sánchez et al. found improved exercise endurance and increased type I muscle fibers in Elavl1-knockout mice (90). These discrepancies highlight the complexity of ELAVL1’s role, and our results, based on whole blood analysis, require further investigation. Interestingly, we observed two expression clusters. *FOXO1* and *ELAVL1* were strongly correlated across all the study groups consistent with their reported interactions , where ELAVL1 stabilizes and posttranscriptionally upregulates FOXO1 (91). A second cluster included *SIRT1*, *SIRT3*, and *DNMT3A* suggesting coordinated regulation. During DNA damage, SIRT1 can recruit DNMTs to promoters to induce methylation and silence damaged genes (92). Furthermore, in the sarcopenic group, we also detected upregulation and a strong correlation between *FOXO1* and *SIRT6* mRNA levels, which potentially may reflect the activation of the SIRT6/FOXO1 axis in the blood cells of sarcopenic patients. The SIRT6-FoxO1 pathway is associated with the process of insulin and lipid metabolism (93, 94). Nonetheless, the role of these pathways in sarcopenia and inflammaging requires further research.

### 4.6 Limitations of the study and future studies

The main limitation of the present research is the sample size in the sarcopenia group. However, this study was set up as a pilot study for a further functional and large-scale study focused on FOXO3A. Additionally, the reliance on transcriptomic analysis without integrating protein-level data and functional outcomes limits the translational relevance of the findings. Future studies should be conducted using a multidimensional biomarker approach to address the multifactorial nature of sarcopenia and validate these results in larger cohorts. We are also aware of the potential impact of factors such as the multi-drug or multi-disease nature of the patients studied. The frailty syndrome group in this study includes patients with frailty but without sarcopenia, while the sarcopenia group consists of patients with sarcopenia alone or with coexisting frailty. This design of the study was intentional, as our primary goal was to focus on biomarkers specific to sarcopenia, acknowledging the common co-occurrence of sarcopenia and frailty. However, this prevents conclusions about sarcopenia occurring independently.

In conclusion, we identify a potential blood marker of sarcopenia, namely, the downregulation of *FOXO3A*, which may be related to inflammation. Moreover, *FOXO3A* appears to correlate with additional clinical parameters such as iron, ESR, and LDH.We also observed the upregulation of *SIRT3* and *DNMT3A* in healthy controls over 50 years old compared to the younger control group. Our study indicates directions for future research on inflammaging, sarcopenia, and aging. Altered metabolic pathways in the older adult population, particularly those involving *DNMT3A*, *SIRT3*, and *FOXO3A*, should be considered during the drug design process. This is crucial, as drugs affecting these pathways may act differently in the elderly population due to altered metabolism, polypharmacy, and potential drug-drug interaction.

## Data Availability

The raw data supporting the conclusions of this article will be made available by the authors, without undue reservation.
