# Supplementary figures and images for: Epigenetic factors and inflammaging: FOXO3A as a potential biomarker of sarcopenia and upregulation of DNMT3A and SIRT3 in older adults
Source: Front Immunol. 2025 Feb 17;16:1467308. doi: 10.3389/fimmu.2025.1467308 (PMC11872893; doi:10.3389/fimmu.2025.1467308)

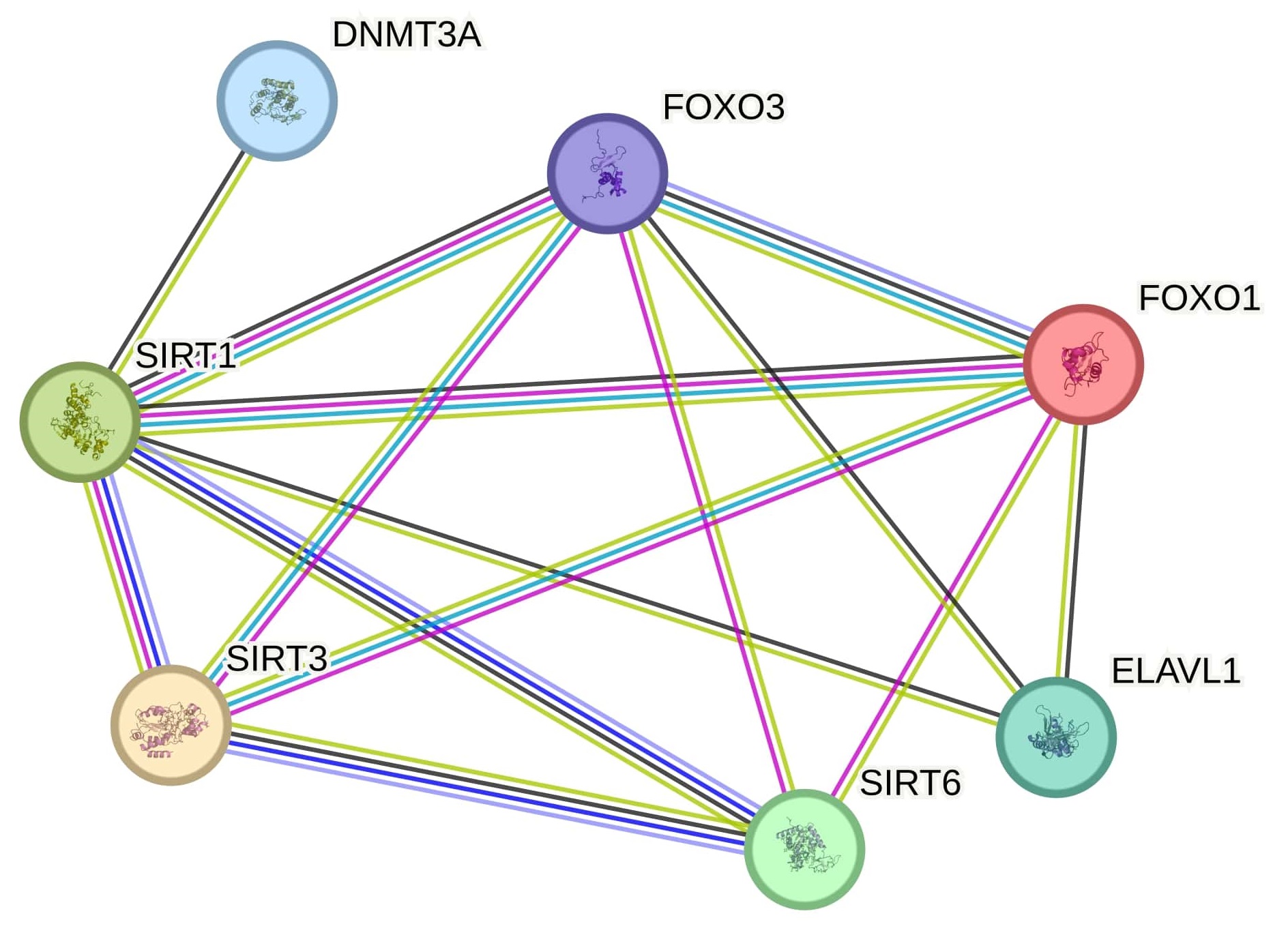

Supplement: Supplementary file 1 [file Image1.jpeg]

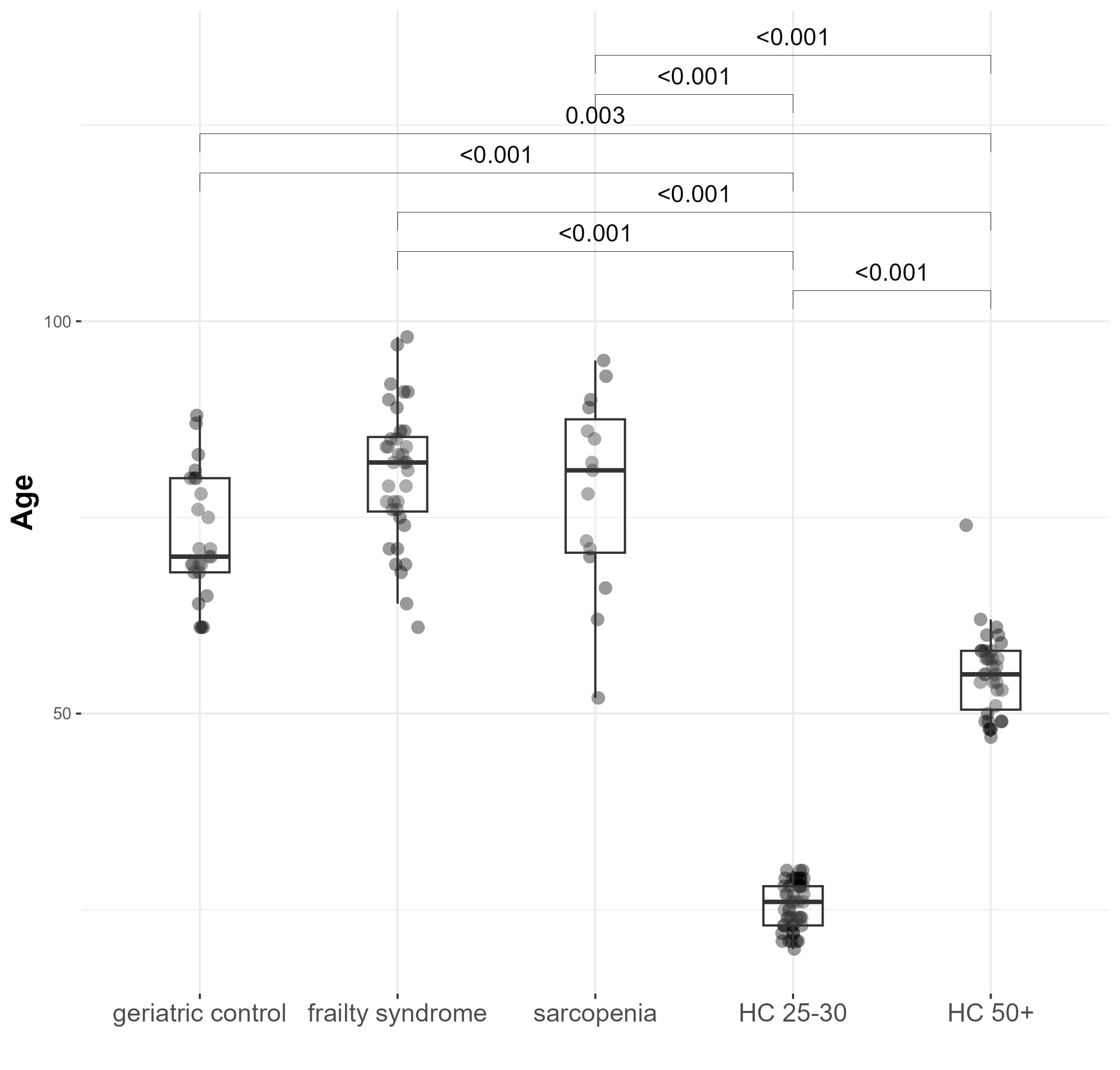

Supplement: Supplementary file 2 [file Image2.jpeg]

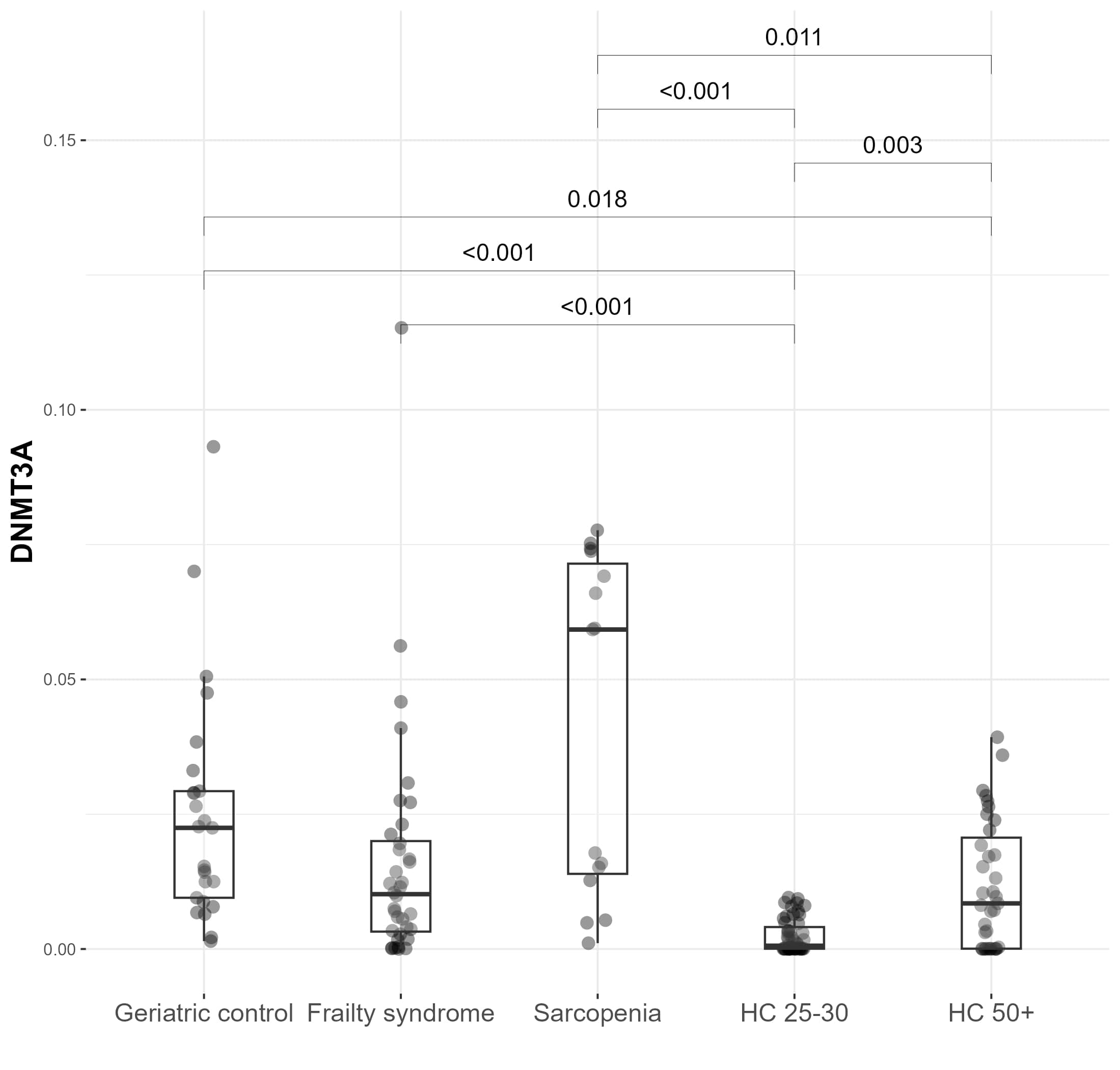

Supplement: Supplementary file 3 [file Image3.jpeg]

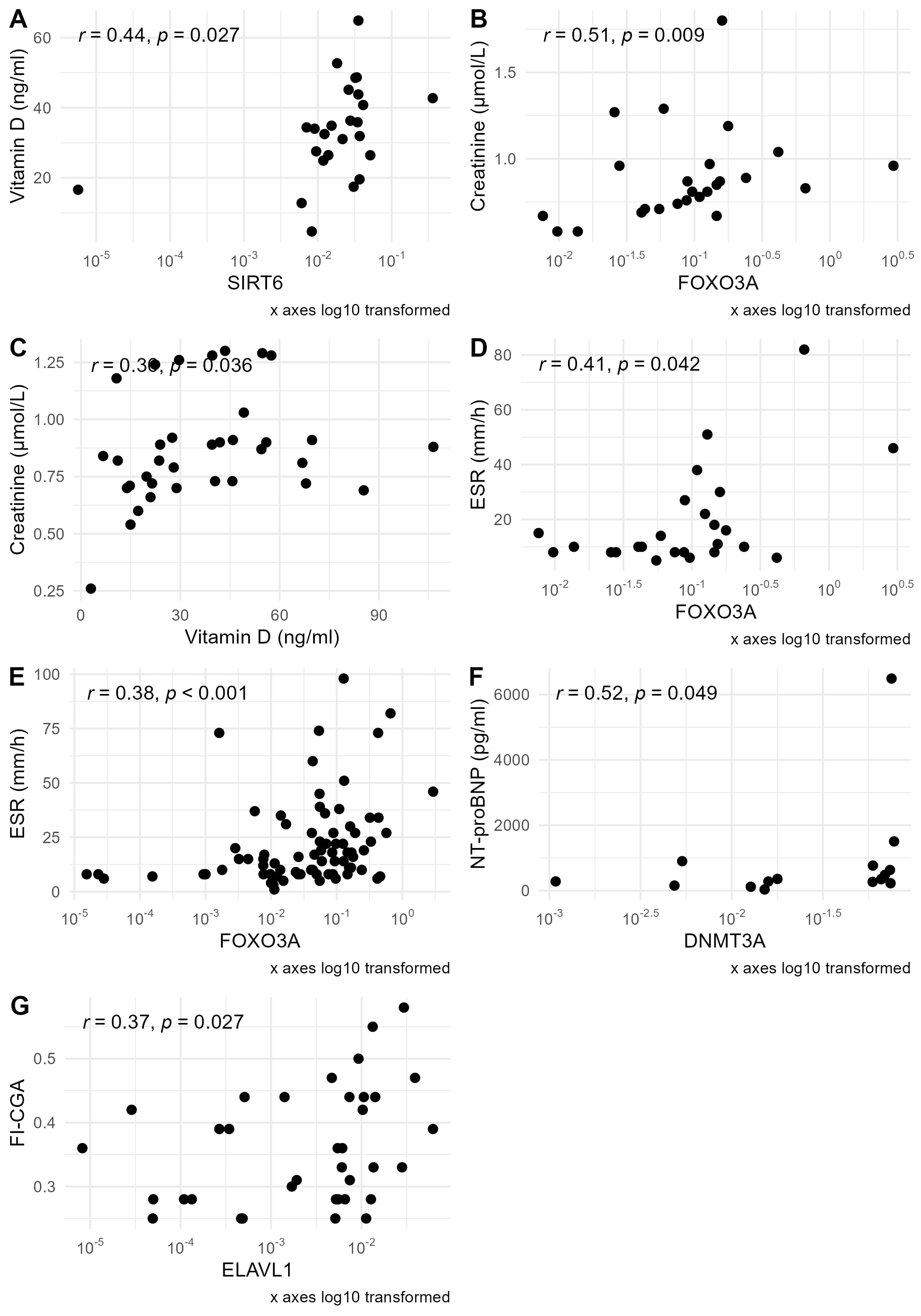

Supplement: Supplementary file 4 [file Image4.jpeg]

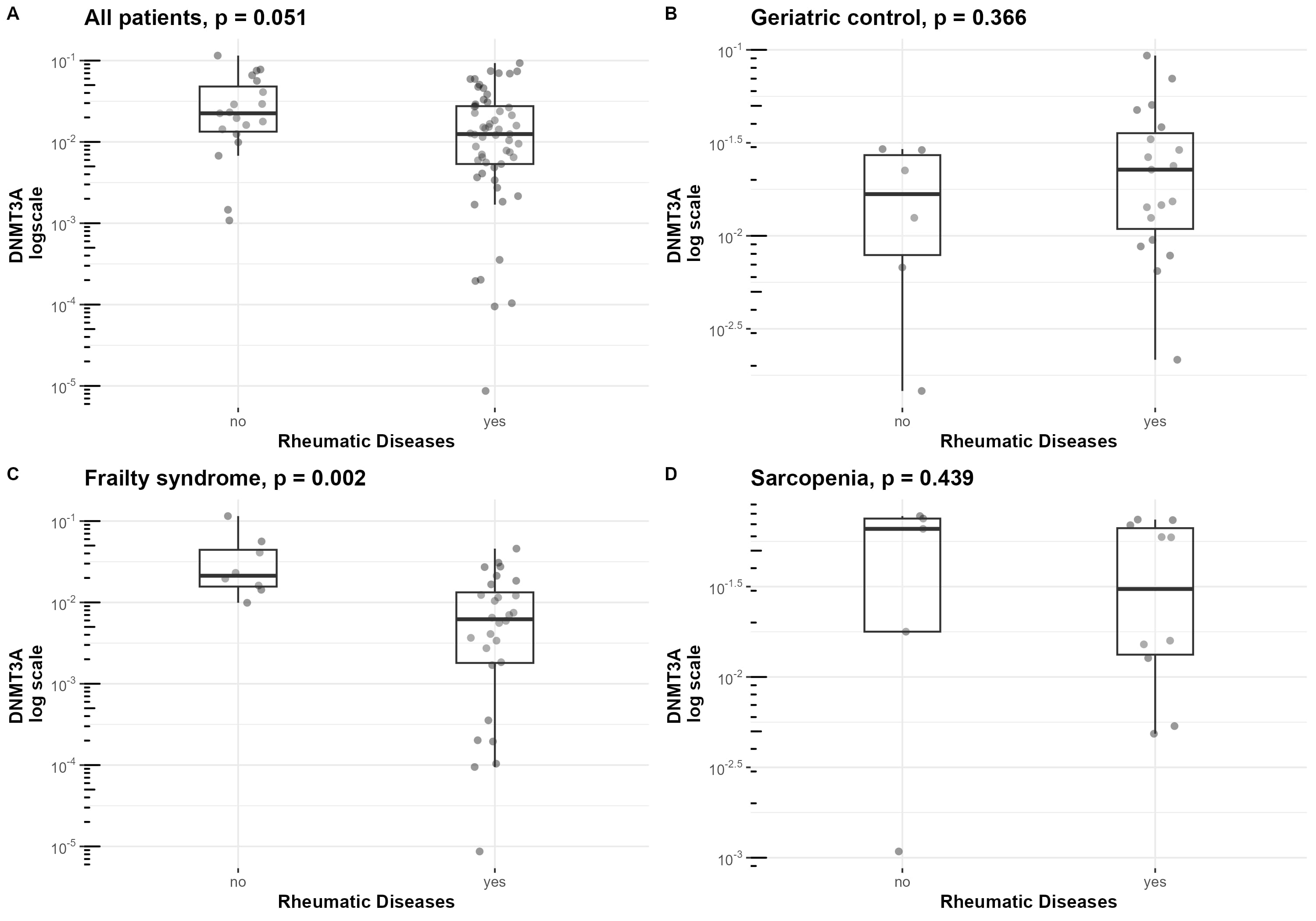

Supplement: Supplementary file 5 [file Image5.jpeg]
