# Supplementary material for: Epigenetic factors and inflammaging: FOXO3A as a potential biomarker of sarcopenia and upregulation of DNMT3A and SIRT3 in older adults
Source: Front Immunol. 2025 Feb 17;16:1467308. doi: 10.3389/fimmu.2025.1467308 (PMC11872893; doi:10.3389/fimmu.2025.1467308)
Supplement: Supplementary file 6 [file Table1.docx]

Supplementary Material

Epigenetic factors and inflammaging. FOXO3A as a potential biomarker of sarcopenia and upregulation of DNMT3A and SIRT3 in older adults.

**Diana Bogucka^1*^, Anna Wajda^1^, Barbara Stypińska^1^, Marcin Jerzy Radkowski^2^, Tomasz Targowski^2^, Ewa Modzelewska^1^, Tomasz Kmiołek^1^, Adam Ejma-Multański^1^, Gabriela Filipowicz^1^, Yana Kaliberda^1^, Ewa Dudek^1^, Agnieszka Paradowska-Gorycka ^1^**

^1^Department of Molecular Biology, National Institute of Geriatrics, Rheumatology and Rehabilitation,02-637 Warsaw, Poland

^2^Department of Geriatrics, National Institute of Geriatrics, Rheumatology and Rehabilitation, 02-637 Warsaw, Poland

*** Correspondence:**Diana Bogucka
diana.bogucka123@gmail.com

# Supplementary Tables

Table S1. Missing data in analyzed groups.

| Analyzed parameter | Missing data  N=168  n (%) | Sarcopenic patients group  N =15  n (%) | Frailty patients group  N = 36  n (%) | Geriatric control group  N = 25  n (%) | Healthy control  25-30 y/o  N = 51  n (%) | Healthy control  50+ y/o  N = 41  n (%) |
| --- | --- | --- | --- | --- | --- | --- |
| Expression of *SIRT1* | 0 (0%) | 0 (0%) | 0 (0%) | 0 (0%) | 0 (0%) | 0 (0%) |
| Expression of *SIRT3* | 0 (0%) | 0 (0%) | 0 (0%) | 0 (0%) | 0 (0%) | 0 (0%) |
| Expression of *DNMT3A* | 0 (0%) | 0 (0%) | 0 (0%) | 0 (0%) | 0 (0%) | 0 (0%) |
| Expression of *FOXO3A* | 4 (2.4%) | 0 (0%) | 0 (0%) | 0 (0%) | 4 (7.8%) | 0 (0%) |
| Expression of *SIRT6* | 4 (2.4%) | 0 (0%) | 0 (0%) | 0 (0%) | 4 (7.8%) | 0 (0%) |
| Expression of *FOXO1* | 5 (3.0%) | 0 (0%) | 0 (0%) | 0 (0%) | 5 (9.8%) | 0 (0%) |
| Expression of *ELAVL1* | 3 (1.8%) | 0 (0%) | 0 (0%) | 0 (0%) | 3 (5.9%) | 0 (0%) |
| Age | 0 (0%) | 0 (0%) | 0 (0%) | 0 (0%) | 0 (0%) | 0 (0%) |
| Gender | 0 (0%) | 0 (0%) | 0 (0%) | 0 (0%) | 0 (0%) | 0 (0%) |
| FI-CGA | 0 (0%) | 0 (0%) | 0 (0%) | 0 (0%) | N/A | N/A |
| TUG (s) | 6 (7.9%) | 0 (0%) | 3 (8.3%) | 3 (12.0%) | N/A | N/A |
| ASMM (kg) | 33 (43.4%) | 0 (0%) | 22 (61.2%) | 11 (44.0%) | N/A | N/A |
| LDH (U/L) | 9 (11.8%) | 7 (46.7%) | 1 (2.8%) | 1 (4.0%) | N/A | N/A |
| CK (U/L) | 2 (2.6%) | 1 (6.7%) | 0 (0%) | 1 (4.0%) | N/A | N/A |
| NYHA Class | 0 (0%) | 0 (0%) | 0 (0%) | 0 (0%) | N/A | N/A |
| Osteopenia | 10 (13.2%) | 0 (0%) | 6 (16.7%) | 4 (16.0%) | N/A | N/A |
| Osteoporosis | 10 (13.2%) | 0 (0%) | 6 (16.7%) | 4 (16.0%) | N/A | N/A |
| Polyarthritis | 0 (0%) | 0 (0%) | 0 (0%) | 0 (0%) | N/A | N/A |
| Degenerative Disc Disease | 0 (0%) | 0 (0%) | 0 (0%) | 0 (0%) | N/A | N/A |
| Any rheumatic disease | 0 (0%) | 0 (0%) | 0 (0%) | 0 (0%) | N/A | N/A |
| Fasting glucose (mg/dl) | 2 (2.6%) | 0 (0%) | 1 (2.8%) | 1 (4.0%) | N/A | N/A |
| Vit. D3 (ng/ml) | 1 (1.3%) | 0 (0%) | 1 (2.8%) | 0 (0%) | N/A | N/A |
| NT-proBNP (pg/ml) | 11 (14.5%) | 0 (0%) | 5 (13.9%) | 6 (24.0%) | N/A | N/A |
| Serum Fe level (mcg/dL) | 44 (57.9%) | 7 (46.7%) | 19 (52.8%) | 18 (72.0%) | N/A | N/A |
| Vit. B12 (pg/mL) | 0 (0%) | 0 (0%) | 0 (0%) | 0 (0%) | N/A | N/A |
| ESR (mm/h) | 0 (0%) | 0 (0%) | 0 (0%) | 0 (0%) | N/A | N/A |
| CRP (mg/dL) | 0 (0%) | 0 (0%) | 0 (0%) | 0 (0%) | N/A | N/A |
| Albumines (g/dl) | 1 (1.3%) | 0 (0%) | 1 (2.8%) | 0 (0%) | N/A | N/A |
| Hemoglobine (g/dl) | 0 (0%) | 0 (0%) | 0 (0%) | 0 (0%) | N/A | N/A |
| Creatinine (µmol/L) | 0 (0%) | 0 (0%) | 0 (0%) | 0 (0%) | N/A | N/A |
| Cholesterol (mg/dL) | 1 (1.3%) | 0 (0%) | 1 (2.8%) | 0 (0%) | N/A | N/A |
| LDL (mg/dL) | 1 (1.3%) | 0 (0%) | 1 (2.8%) | 0 (0%) | N/A | N/A |
| Triglycerides (mm/l) | 1 (1.3%) | 0 (0%) | 1 (2.8%) | 0 (0%) | N/A | N/A |
| BMI (kg/m2) | 1 (1.3%) | 0 (0%) | 1 (2.8%) | 0 (0%) | N/A | N/A |

FI-CGA - Frailty Index Based on a Comprehensive Geriatric Assessment; TUG - Timed up-and-go scale (mobility scale); ASMM- appendicular skeletal muscle mass; LDH- Lactate Dehydrogenase; CK ‑ creatine phosophokinase; NYHA class - New York Heart Association Functional Classification; NT‑proBNP - N-terminal pro B-type natriuretic peptide; Fe- iron; ESR- erythrocyte sedimentation rate; CRP- C-reactive protein; LDL - low-density lipoprotein; BMI – body mass index; y/o – years old

Table S2. Coefficient of correlation between gene expression level normalized to reference gene and age.

| Age (rho) | Geriatric control group | Frailty patients group | Sarcopenic patients group | Healthy controls |
| --- | --- | --- | --- | --- |
| *SIRT1* | 0.10 | 0.18 | -0.03 | 0.14 |
| *SIRT3* | 0.21 | 0.23 | -0.21 | 0.11 |
| *DNMT3A* | 0.27 | 0.23 | -0.12 | 0.21 |
| *FOXO3A* | -0.02 | 0.13 | -0.09 | 0.15 |
| *SIRT6* | 0.11 | 0.26 | 0.09 | -0.22* |
| *FOXO1* | 0.34 | 0.21 | -0.10 | -0.11 |
| *ELAVL1* | 0.23 | 0.28 | 0.07 | -0.09 |

Healthy patients from Healthy control 25-30 and 50+ groups were analyzed together. Significant correlations were marked with: *<0.05; **<0.01; ***<0.001.

# Supplementary Figures

Figure S1. Network of genes association based on the Strings tool.

Types of interactions: Cyan-from curated databases; Blue-gene co-occurrence; Pink-experimentally determined; Light green-from text mining; Light blue-protein homology; Black-coexpression.

Figure S2. Age differences between studied groups. Data are presented as boxplot with median and range. Only statistically significant differences are shown.

HC25-30 - Healthy control 25-30 years old, HC50+ - Healthy control over 50 years old,

Figure S3. *DNMT3A* mRNA level in each analyzed group normalized to reference gene. Linear scale. Data are presented as boxplot with median and range. Only statistically significant differences are shown.

HC25-30 - Healthy control 25-30 years old, HC50+ - Healthy control over 50 years old.

Figure S4. Correlations of clinical parameters. mRNA levels of studied genes were normalized to reference gene and are presented on logarithmic scale. **(A)** Correlation of vitamin D level with *SIRT6* mRNA level analyzed in geriatric control group. **(B)** Correlation of creatinine level with *FOXO3A* mRNA level analyzed in geriatric control group. **(C)** Correlation of serum creatinine concentration with vitamin D level analyzed in frailty syndrome group. **(D)** Correlation of ESR level with *FOXO3A* mRNA level analyzed in geriatric group. **(E)** Correlation of ESR level with *FOXO3A* mRNA level analyzed in group of all patients together. **(F)** Correlations of NT-proBNP with *DNMT3A* mRNA level analyzed in sarcopenic patients group. **(G)** Correlation of FI-CGA value with *ELAVL1* mRNA level analyzed in frailty syndrome group.

Figure S5. Differences in *DNMT3A* mRNA level normalized to reference gene in groups of patients with or without any rheumatic diseases. Data are presented as boxplot with median and range. *p<0.05 statistical significance.

no: no rheumatic diseases, yes: any rheumatic disease, **(A)** All three hospitalized groups combined (Geriatric control group, Frailty syndrome group, Sarcopenic group), **(B)** Geriatric control group, **(C)** Frailty syndrome group, **(D)** Sarcopenic group.
